# The Triprenylated Anthranoid Ferruginin A, a Promising Scaffold for the Development of Novel Antibiotics against Gram-Positive Bacteria

**DOI:** 10.3390/antibiotics11010084

**Published:** 2022-01-11

**Authors:** Bruno Casciaro, Francesca Ghirga, Floriana Cappiello, Valeria Vergine, Maria Rosa Loffredo, Silvia Cammarone, Elena Puglisi, Carola Tortora, Deborah Quaglio, Mattia Mori, Bruno Botta, Maria Luisa Mangoni

**Affiliations:** 1 Department of Biochemical Sciences, Laboratory Affiliated to Istituto Pasteur Italia-Fondazione Cenci Bolognetti, Sapienza University of Rome, 00185 Rome, Italy; bruno.casciaro@uniroma1.it (B.C.); floriana.cappiello@uniroma1.it (F.C.); mariarosa.loffredo@uniroma1.it (M.R.L.); elena.puglisi@uniroma1.it (E.P.); 2 Department of Chemistry and Technology of Drugs, “Department of Excellence 2018–2022”, Sapienza University of Rome, 00185 Rome, Italy; francesca.ghirga@uniroma1.it (F.G.); valeria.vergine@uniroma1.it (V.V.); silvia.cammarone@uniroma1.it (S.C.); carola.tortora@uniroma1.it (C.T.); bruno.botta@uniroma1.it (B.B.); 3 Department of Biotechnology, Chemistry and Pharmacy, “Department of Excellence 2018–2022”, University of Siena, 53100 Siena, Italy; mattia.mori@unisi.it

**Keywords:** antibiotic-resistance, antimicrobial activity, Gram-positive bacteria, biofilm, anthranoid, natural products

## Abstract

In today’s post-antibiotic era, the search for new antimicrobial compounds is of major importance and nature represents one of the primary sources of bioactive molecules. In this work, through a cheminformatics approach, we clustered an in-house library of natural products and their derivatives based on a combination of fingerprints and substructure search. We identified the prenylated emodine-type anthranoid ferruginin A as a novel antimicrobial compound. We tested its ability to inhibit and kill a panel of Gram-positive and Gram-negative bacteria, and compared its activity with that of two analogues, vismione B and ferruanthrone. Furthermore, the capability of these three anthranoids to disrupt staphylococcal biofilm was investigated, as well as their effect on the viability of human keratinocytes. Ferruginin A showed a potent activity against both the planktonic and biofilm forms of Gram-positive bacteria (i.e., *Staphylococcus aureus* and *S. epidermidis*) and had the best therapeutic index compared to vismione B and ferruanthrone. In conclusion, ferruginin A represents a promising scaffold for the further development of valuable antimicrobial agents.

## 1. Introduction

The discovery of penicillin and the subsequent use of antibiotics in the treatment of bacterial infections has drastically changed human lifespan, increasing it by about 23 years [1]. However, the golden age of antibiotics is at the end; fewer and fewer compounds are being developed, while the onset of multidrug-resistant strains is increasing, making classic therapies mostly useless [2]. There is therefore an urgent need to discover new compounds capable of overcoming antimicrobial resistance (AMR). Although it is difficult to quantify and to predict the number of deaths per year due to AMR, it is estimated that in 2050 it will be 10 million [3]. These numbers are alarming, considering that many microorganisms can transition from a planktonic lifestyle to a sessile form, called biofilm. In biofilm communities, bacterial cells are surrounded by extra-cellular polymeric substances and DNA, which protect them from the antibiotic action, making their eradication even more difficult [4]. As the history of penicillin has taught us, nature is one of the primary sources of bioactive compounds [5,6]. A unique high-diversity library composed of around 1000 individual natural products, isolated mainly from indigenous plants collected in biodiversity-rich countries especially in the tropics and subtropics, is stored at the Organic Chemistry Laboratory of the Department of Chemistry and Technology of Drugs (Sapienza University of Rome, Rome, Italy) [7]. Complementarity between biogenetic enzymes and eukaryotic protein domains is the general molecular basis for the prominence of natural products in biomedical research and drug discovery [8]. Indeed, the structural and chemical diversity of natural products cannot be matched by any synthetically-based corporate screening library, and they remain the single most productive source of leads in modern drug discovery projects, often providing chemical structures as useful platforms for the development of drugs, or for the understanding of biological processes [9]. In recent years, the library has been enlarged with semi-synthetic and synthetic derivatives of several natural compounds, including up to 2000 components. Computer-aided methods have been efficiently exploited to screen this in-house library towards specific biological targets, and several hits and leads have been identified thanks to the wide range of pharmacophores and the high scaffold diversity of the library, which is continuously enlarged with compounds not available from other commercial or literature sources [10,11,12,13]. In the present study, compounds of the in-house library were clustered based on a combination of fingerprints and substructure search through a cheminformatics approach previously established [14,15], with the aim of grouping the library members into homogeneous chemical clusters. The representative compounds of the most populated clusters (**1**–**25**) have been screened for their strength in inhibiting and killing bacterial cells, as well as for their antibiofilm and cytotoxic activities. Interestingly, compound **7** showed a potent anti-Gram-positive activity, especially against the biofilm forms of *Staphylococcus aureus* and *S. epidermidis*, along with the better biological profile. Overall, our findings suggest molecule **7** as a novel antimicrobial candidate for further investigation and development.

## 2. Results

### 2.1. Screeening of Compounds: Structural Characterization and Preliminary Antibacterial Profile

Our in-house library is a valid source of chemotypes for the modulation of biomolecular targets, and it has been successfully screened in silico and in vitro for the identification of hit and lead compounds in previous early-stage drug discovery projects [7].

To focus experimental efforts on a relatively low number of molecules, and to explore the chemical and scaffold space of the library as much as possible, a cheminformatics approach was employed to identify new potential chemotypes of antibacterial agents. To this end, a diversity-oriented random selection (DORS) of compounds was performed by means of a clustering algorithm, which relies on a combination of fingerprints and common substructure search to group compounds endowed with chemical similarity index within a given threshold [14]. Thanks to the algorithm, compounds of the library were grouped, and the representative molecules of the most abundant clusters (**1**–**25**) were selected for biological assays (Table 1).

Compounds **1**–**25** were screened in vitro against reference Gram-negative (i.e., *Escherichia coli*) and Gram-positive (i.e., *S. aureus* and *S. epidermidis*) bacterial strains by evaluating their capability of inhibiting microbial growth through the inhibition zone assay. All compounds were not active against *E. coli* (data not shown), while only few of them were active against the Gram-positive bacterial strains (Table 2).

According to the literature, gallic acid (**6**) showed a significant antimicrobial activity against *S. aureus*, while usnic acid (**17**) and 2′,4-hydroxy-4′-methoxy-chalcone (**18**) showed activity against both *S. aureus* and *S. epidermidis* [52,53,54].

In addition, compound **7**, ferruginin A, showed a good activity against both strains, with an inhibition zone ranging from 0.55 to 0.62 cm. Considering the limited number of studies performed on its biological activity to date, we decided to characterize its antimicrobial and cytotoxic activity. Ferruginin A is a prenylated emodine-type anthranoid, a polyphenols subclass, isolated from *Vismia baccifera* var. *ferruginea* and *Vismia decipiens* [22]. To thoroughly investigate the antibacterial activity of the emodine-type anthranoid scaffold, we also tested two other structurally-related compounds, ferruanthrone (**26**) and vismione B (**27**), reported in Table 3.

These anthranoids share the same biogenetic pathway and were isolated from the same plant. Compound **26** is classified as one of the unique compounds of the library, i.e., not available from other commercial or literature sources. It differs from compound **7** for the anthrone scaffold, a tricyclic aromatic ketone, and the position of the prenyl groups [22]. Compound **27** is a pre-anthraquinone non substituted with a γ, γ-dimethylallyl chain cyclized to form a chromane ring in pace of the C-prenyl group [55].

### 2.2. Antimicrobial Activity of Compounds ***7***, ***26***, and ***27***

The antimicrobial activity of **7**, **26**, and **27** was evaluated against different Gram-negative and Gram-positive bacterial strains and a yeast by determining their minimum inhibitory concentrations (MIC) by the microdilution assay in broth. After an incubation of about 18 h, the concentration of compounds able to totally inhibit microbial growth was detected and is reported in Table 4.

Compounds **26** and **27** were inactive against all the strains tested, with the only exception being *B. megaterium* Bm11 (MIC = 256 μM). Compound **7** was inactive against *C. albicans* and Gram-negative strains. In comparison, according to the results of the inhibition zone assay (Table 2), it showed a potent activity against Gram-positive microorganisms with MIC values of 8 μM against *B. megaterium*, 16 μM against *S. epidermidis,* and 64 μM against *S. aureus*.

**Figure 1:**
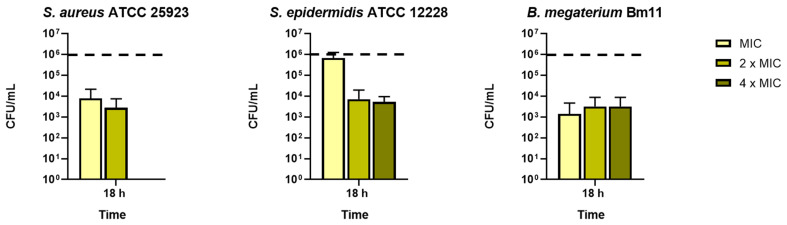
Bactericidal activity of compound **7** at its MIC, 2 × MIC, and 4 × MIC evaluated after 18 h of treatment by CFU counting. The values are the mean ± the standard error of the mean (SEM) obtained from at least three independent experiments. The dotted line indicates the initial number of bacterial cells (1 × 10^6^ CFU/mL).

As **7** was the only active compound, we investigated whether its antimicrobial activity was due to a bacteriostatic or bactericidal effect. To this aim, aliquots from the wells corresponding to MIC, 2 × MIC, and 4 × MIC were plated on agar plates for the counting of colony-forming units (CFU). As reported in Figure 1, compound **7** provoked about a 2-log reduction of viable *S. aureus* cells within 18 h treatment at its MIC and 2 × MIC and a total killing at its 4 × MIC. Differently, against *S. epidermidis*, **7** showed a bactericidal effect (about 2-log reduction of viable cells) at its 2 × MIC and 4 × MIC. Compound **7** also displayed the same antimicrobial efficacy against *B. megaterium* at all the concentrations tested.

### 2.3. Antibiofilm Activity

Staphylococci are recognized as the most frequent etiological agents of biofilm-associated infections [56], and for this reason, compounds **7**, **26**, and **27** were tested for their ability to eradicate preformed biofilm of *S. aureus* and *S. epidermidis* by evaluating the biofilm viability through the 3-(4,5-dimethylthiazol-2-yl)-2,5-diphenyltetrazolium bromide (MTT)-based assay.

**Figure 2:**
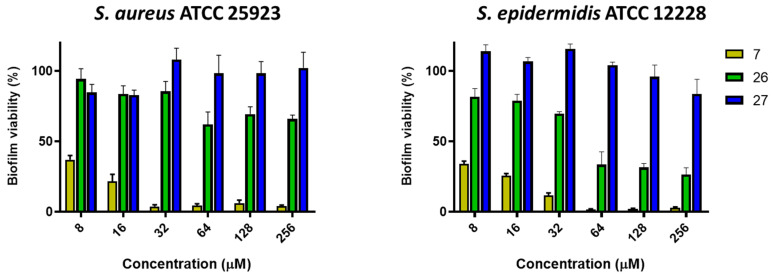
Activity of compounds **7**, **26**, and **27** against preformed *S. aureus* and *S. epidermidis* biofilms, evaluated after 2 h of treatment, compared to the untreated control cells, using the MTT assay. The values are the means ± SEM of triplicates of three independent experiments.

As reported in Figure 2, **27** was inactive (100% biofilm viability) against preformed biofilms of both strains at all concentrations. Differently, **26** showed only a weak activity at higher concentrations ranging from 64 to 256 μM against the *S. aureus* biofilm, while a more pronounced activity (about 60% of killing) was recorded against the *S. epidermidis* biofilm at the same concentrations. Considering its MIC values against both strains (i.e., >256 μM), these results have indicated a different activity of compound **26**; despite it was not able to completely inhibit microbial growth after 18 h, it caused a significant reduction in the amount of viable biofilm cells within a short time (i.e., 2 h of treatment). Compound **7** revealed to be the most efficacious molecule causing more than 90% biofilm killing at the concentration range between 32 and 256 μM, and about 60% killing at lower concentrations of 16 and 8 μM, against both strains.

**Figure 3:**
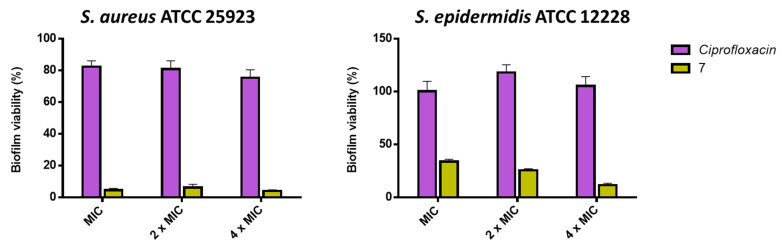
Activity of compound **7** and ciprofloxacin at MIC, 2 × MIC, and 4 × MIC against preformed *S. aureus* and *S. epidermidis* biofilms, after 2 h of treatment, compared to the untreated control cells, using the MTT assay. The values are the means ± SEM of the triplicates of three independent experiments.

To better estimate the extent of the anti-biofilm activity of compound **7**, we then compared our findings with the activity of a conventional antibiotic (i.e., ciprofloxacin) against the preformed biofilm of *S. aureus* ATCC 25923 and *S. epidermidis* ATCC 12228 at MIC, 2 × MIC, and 4 × MIC. As reported in Figure 3, ciprofloxacin was inactive against the *S. epidermidis* biofilm, while only 20% killing of biofilm cells (~80% of biofilm viability) was detected at all concentrations tested against *S. aureus*.

### 2.4. Cytotoxicity

**Figure 4:**
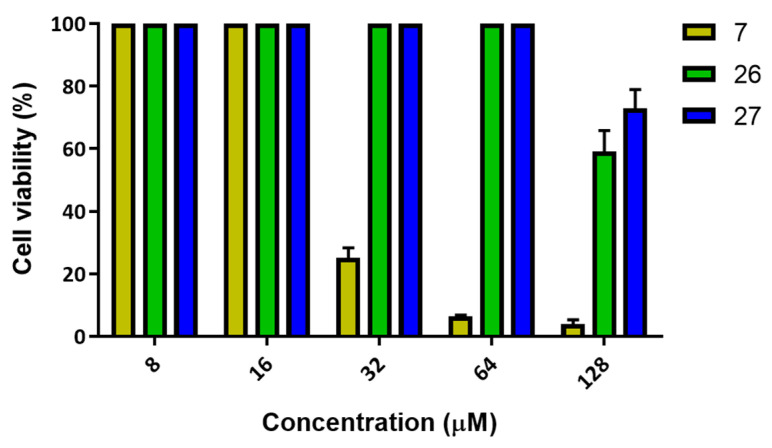
HaCaT cells viability after 24 h treatment with compounds **7**, **26**, and **27** compared to the untreated control cells, as determined by the MTT assay. Data are expressed as the mean of three independent experiments ± SEM.

To investigate any potential harmful action of compounds **7**, **26**, and **27** on host tissues (a crucial aspect for clinical antimicrobial applications of drugs), we evaluated their effect on the viability of human immortalized HaCaT cells, the major cell type of human skin [57,58], using the MTT assay. After 24 h of treatment, compounds **26** and **27** did not induce any toxicity up to a concentration of 64 μM; at 128 μM, cell viability was approximately 60% and 72% for **26** and **27**, respectively (Figure 4). A different trend was shown for compound **7**, which was not toxic at 8 μM and 16 μM; however, incubation of HaCaT cells with increasing concentrations of this compound led to about 25% cell viability at 32 μM and less than 10% at 64 μM and 128 μM (Figure 4). All three compounds were strongly toxic at 256 μM (data not shown).

Based on the in vitro results of the antibiofilm and cytotoxic activities, we calculated the lethal concentration causing 50% cell death (LC_50_) and the minimum effective dose for 50% biofilm killing (ABC_50_) to evaluate the potential therapeutic index (TI) of the most active compounds (i.e., **7** and **26**; Table 5).

As the TI value was calculated as the ratio between the toxic concentrations and the effective concentrations, the higher the value, the better the biological profile. Although compound **7** showed cytotoxicity compared to **26**, its TI values (i.e., 25.81–6.19) were higher than those of **26**, underlining compound **7** as the most promising new antibiofilm molecule.

## 3. Discussion

The demand for new antibiotic compounds is of major importance in the post-antibiotic era we are currently living. Investments by pharmaceutical companies are often directed to projects aimed at producing drugs for the treatment of chronic diseases that can bring greater revenues than antimicrobials [59]. However, microbial infections remain a challenge to keep under constant control. Indeed, the European Centre for Disease Prevention and Control has conservatively estimated that, in Europe alone, AMR can cause additional annual cost to health care systems of at least €1.5 billion [60].

Considering that biofilm formation is involved in 65–80% of bacterial infections in humans, new antibiotic compounds must have the ability to kill not only the planktonic but also the sessile form of microorganisms [56]. Conventional antibiotics are usually not efficacious against biofilms. Therefore, to prevent the onset of resistant and persistent bacterial cells, it would ideal to identify a molecule able to disrupt sessile bacterial communities within a short time. Here, through a clustering algorithm, we obtained a set of molecules that were firstly screened for their antimicrobial activity. Ferruginin A (**7**) was found to be the most interesting molecule, due to its anti-Gram-positive activity and the poor information available in the literature. The molecule was isolated in the 1979 from *Vismia baccifera* var. *ferruginea* [22], as well as from other *Vismia* species [61]. Over the years, ferruginin A (**7**) was also isolated from the leaves of *Harungana madagascariensis;* Tankeo and coworkers tested it, together with betulinic acid, madagascin, and Kaempferol-3-O-β-D-gluco-pyranoside, for its antimicrobial activity using ciprofloxacin as an antibiotic control, but also against a panel of Gram-negative bacteria (e.g., *Enterobacter cloacae*, *Providencia stuartii*, and *Klebsiella pneunomaniae*) [62,63]. Considering that Staphylococci, especially *S. aureus* and *S. epidermidis*, are common inhabitants of the human skin microbiota, as well as being the major microorganisms responsible for hospital-acquired infections, we characterized the capability of ferruginin A (**7**) to kill the biofilm cells of these pathogens [64]. Compound **7** showed a potent antimicrobial activity against Gram-positive bacteria (MIC values ranging from 8 to 64 μM) and a bactericidal rather than a bacteriostatic effect, as indicated by its ability to provoke a significant CFU reduction. In addition, compound **7** was found to possess a potent antibiofilm activity against both *S. aureus* and *S. epidermidis*, with an ABC_50_ of 1.08 and 4.5 μM, respectively. In comparison to the two related compounds ferruanthrone (**26**) and vismione B (**27**), ferruginin A (**7**) had the higher antibiofilm activity, but also the higher cytotoxicity against HaCaT cells. Despite this, its biological profile (evaluated as TI; Table 5) was better than compound **26** (25.81–6.19 vs. 0.26–2.65, respectively). It is worth noting that the antibiofilm activity was obtained only after 2 h of treatment, while ciprofloxacin, used as a control, was totally inactive against both staphylococcal biofilms within such short time. This fast kinetics is of high importance also for destroying biofilms associated with medical devices. The use of medical devices is rapidly increasing and, despite the multiple precautionary sterilization measures that are being followed in hospitals, staphylococcal colonization on these devices still happens quite frequently [65,66]. Although compound **7** exhibits cytotoxicity at concentrations greater than 32 μM, advances in nanotechnologies would likely help to overcome its noxious effects upon incorporation into appropriate nanoparticulate systems. In addition, it could be developed as an antibiofilm agent, only for disinfection of medical equipment and surgical tools. Furthermore, as even small changes in the chemical structure of ferruginin analogs (**26** and **27**) are sufficient to lead to a substantial modification of their activity /toxicity, this confirms ferruginin A as a novel promising scaffold for further development of valuable antimicrobial agents.

## 4. Materials and Methods

### 4.1. In-House Library Clustering

The algorithm has been described previously [14], and it is inspired by the work published by Stahl and Mauser [15]. Briefly, the custom Python script first performs a preliminary clustering based on maccs166 fingerprints, grouping compounds with a Tanimoto similarity equal or higher than 0.8. Then, isolated compounds are assigned to pre-formed clusters based on substructure comparison, if they have a substructure matching equal or higher than 0.85 according to the Raymond cutoff. Compounds not matching these criteria are classified as singletons and are not included in any of the existing clusters.

### 4.2. Chemistry

All of the tested compounds (namely, **1**–**27**) are known structures belonging to our in-house library of natural products. The chemical identity of the compounds was assessed by re-running nuclear magnetic resonance spectroscopy (NMR) experiments and were proven to agree with the literature data reported below for each compound. The purity of all compounds, checked by reversed-phase high performance liquid chromatography (HPLC), was always higher than 95%.

Compound **1** (Ibogaine or (6R,7S,11S)-7-ethyl-2-methoxy-6,6a,7,8,9,10,12,13-octahydro-5H-6,9- methanopyrido[1′,2′:1,2]azepino[4,5-b]indole) showed NMR spectra identical to those reported in the literature.

Compound **2** (Serotonin or 3-(2-aminoethyl)-1H-indol-5-ol) was purchased from Sigma-Aldrich (CAS: 50-67-9, St. Louis, MO, USA) and was used without further purification.

Compound **3** (Caffeine or 1,3,7-trimethyl-3,7-dihydro-1H-purine-2,6-dione) was purchased from Sigma-Aldrich (CAS: 58-08-2, St. Louis, MO, USA) and was used without further purification.

Compound **4** (Veratric acid or 3,4-dimethoxybenzoic acid) was purchased from Sigma-Aldrich (CAS: 93-07-2, St. Louis, MO, USA) and was used without further purification.

Compound **5** (Cinnamic acid) was purchased from Sigma-Aldrich (CAS: 140-10-3, St. Louis, MO, USA) and was used without further purification.

Compound **6** (Gallic acid or 3,4,5-trihydroxybenzoic acid) was purchased from Sigma-Aldrich (CAS: 149-91-7, St. Louis, MO, USA) and was used without further purification.

Compound **7** (Ferruginin A or 4,5,10-trihydroxy-7-methyl-1,1,6-tris(3-methylbut-2-enyl)anthracen-2-one) showed NMR spectra identical to those reported in the literature.

Compound **8** (Trachyphone or 4,4’,5,5’-tetrahydroxy-2,2’-dimethoxy-3,3’,7,7’-tetramethyl-[1,1’-bianthracene]-9,9’,10,10’-tetraone) showed NMR spectra identical to those reported in the literature.

Compound **9** (Aloin or 1,8-dihydroxy-3-(hydroxymethyl)-10-(3,4,5-trihydroxy-6-(hydroxymethyl)tetrahydro-2H-pyran-2-yl)anthracen-9(10H)-one) showed NMR spectra identical to those reported in the literature.

Compound **10** (Deguelin or (7aS,13aS)-9,10-dimethoxy-3,3-dimethyl-13,13a-dihydro-3H-pyrano[2,3-c:6,5-f’]dichromen-7(7aH)-one) was purchased from Sigma-Aldrich (CAS: 522-17-8, St. Louis, MO, USA) and used without further purification.

Compound **11** (Pongapin or 2-(benzo[d][1,3]dioxol-5-yl)-3-methoxy-4H-furo[2,3-h]chromen-4-one) showed NMR spectra identical to those reported in the literature.

Compound **12** (7-hydroxy-flavone or 7-hydroxy-2-phenyl-4H-chromen-4-one) showed NMR spectra identical to those reported in the literature.

Compound **13** (Glabrescione B or 3-(3,4-bis((3-methylbut-2-en-1-yl)oxy)phenyl)-5,7-dimethoxy-4H-chromen-4-one) showed NMR spectra identical to those reported in the literature.

Compound **14** (Osajin or 5-hydroxy-3-(4-hydroxyphenyl)-8,8-dimethyl-6-(3-methylbut-2-en-1-yl)-4H,8H-pyrano[2,3-f]chromen-4-one) showed NMR spectra identical to those reported in the literature.

Compound **15** (Sakuranetin or 5-hydroxy-2-(4-hydroxyphenyl)-7-methoxychroman-4-one) showed NMR spectra identical to those reported in the literature.

Compound **16** (Clusiacitran B or (3-hydroxy-6,6,9-trimethyl-6a,7,8,9,10,10a-hexahydro-6H-1,9-epoxybenzo[c]chromen-2-yl)(phenyl)methanone) showed NMR spectra identical to those reported in the literature.

Compound **17** (Usnic acid or (R)-1,1’-(1,7,9-trihydroxy-8,9b-dimethyl-3-oxo-3,9b-dihydrodibenzo[b,d]furan-2,6-diyl)bis(ethan-1-one) showed NMR spectra identical to those reported in the literature.

Compound **18** (2’,4-hydroxy-4’-methoxy-chalcone or (E)-1-(2-hydroxy-4-methoxyphenyl)-3-(4-hydroxyphenyl)prop-2-en-1-one) showed NMR spectra identical to those reported in the literature.

Compound **19** (4,4’-dimethoxy-chalcone or (E)-1,3-bis(4-methoxyphenyl)prop-2-en-1-one) showed NMR spectra identical to those reported in the literature.

Compound **20** (2-hydroxy-dihydrochalcone or 3-(2-hydroxyphenyl)-1-phenylpropan-1-one) showed NMR spectra identical to those reported in the literature.

Compound **21** (Xanthotoxin or 9-methoxy-7H-furo[3,2-g]chromen-7-one) showed NMR spectra identical to those reported in the literature.

Compound **22** (Columbianetin or (S)-8-(2-hydroxypropan-2-yl)-8,9-dihydro-2H-furo[2,3-h]chromen-2-one) showed NMR spectra identical to those reported in the literature.

Compound **23** (Borneol or (2S)-1,7,7-trimethylbicyclo[2.2.1]heptan-2-ol) showed NMR spectra identical to those reported in the literature.

Compound **24** (Ursolic acid or (1S,2R,4aS,6aS,6bR,8aR,10S,12aR,12bR,14bS)-10-hydroxy-1,2,6a,6b,9,9,12a-heptamethyl-1,3,4,5,6,6a,6b,7,8,8a,9,10,11,12,12a,12b,13,14b-octadecahydropicene-4a(2H)-carboxylic acid) was purchased from Sigma-Aldrich (CAS: 77-52-1, St. Louis, MO, USA) and was used without further purification.

Compound **25** (Bixin or (2E,4E,6E,8E,10E,12E,14E,16E,18E)-20-methoxy-4,8,13,17-tetramethyl-20-oxoicosa-2,4,6,8,10,12,14,16,18-nonaenoic acid) showed NMR spectra identical to those reported in the literature.

Compound **26** (Ferruanthrone or 1,6,8-trihydroxy-3-methyl-2,4,7-tris(3-methylbut-2-en-1-yl)anthracen-9(10H)-one) showed NMR spectra identical to those reported in the literature.

Compound **27** (Vismione B or 9,12-dihydroxy-5-methoxy-2,2,9-trimethyl-2,8,9,10-tetrahydro-11H-naphtho[2,3-h]chromen-11-one) showed NMR spectra identical to those reported in the literature.

### 4.3. Materials, Bacterial Strains and Cell Line

All reagents and antibiotics used were purchased from Sigma-Aldrich (St. Luis, MO, USA).

The bacterial strains used in the antimicrobial assays were, for Gram-negative, *E. coli* ATCC 25922 and *P. aeruginosa* ATCC 27853, and for Gram-positive, *B. megaterium* Bm11, *S. aureus* ATCC 25923, *S. epidermidis* ATCC 12228, and the yeast *C. albicans* ATCC 24433.

HaCaT cells were purchased from AddexBio (San Diego, CA, USA) and were cultured in Dulbecco’s modified Eagle’s medium supplemented with 4 mM glutamine (DMEMg), 10% heat-inactivated fetal bovine serum (FBS), and 0.1 mg/mL of penicillin and streptomycin at 37 °C and 5% CO_2_, in 25 cm^2^ or 75 cm^2^ flasks.

### 4.4. Antibacterial Screening: Inhibition Zone Assay

To screen the library for any antimicrobial activity, we tested all compounds against a reference Gram-negative (i.e., *E. coli*) and two Gram-positive bacterial strains (i.e., *S. aureus* and *S. epidermidis*) through the inhibition zone assay. Bacteria were grown at 37 °C in Luria−Bertani broth (LB) with gentle shaking until reaching an optical density (O.D.) of 0.8 at 590 nm. Then, the bacterial culture was diluted 1:2000 and plated in LB-agarose plates, and aliquots of 3 μL of each compound (5 mM) were loaded into holes previously made in the agarose plates. The plates were incubated overnight, and the diameters of the inhibition zone were measured and are reported in Table 2.

### 4.5. Antimicrobial Assays

The minimum inhibitory concentrations were determined by the microdilution assay in a 96-well plate. Aliquots of 50 μL of bacterial suspension in Mueller–Hinton broth (MH) in mid-log phase (at a concentration of 2 × 10^6^ cells/mL) were added to 50 μL MH containing serial dilutions of the compounds (in a concentration range of 2–256 μM). The controls were vehicle-treated cells. After an incubation time of 16 h at 37 °C, the MIC was defined as the lowest concentration causing 100% visible inhibition of microbial growth. To determine the bactericidal activity of the tested compounds, aliquots from the wells corresponding to the MIC, 2 × MIC, and 4 × MIC were withdrawn and plated onto agar plates for colony forming unit (CFU) counting.

For the antibiofilm activity, *S. aureus* and *S. epidermidis* were grown as reported above; then aliquots of 100 µL of bacteria in LB (at a concentration of 1 × 10^6^ CFU/mL) were dispensed into the wells of a 96-multiwell plate, which was incubated for 20 h at 37 °C to allow for biofilm formation. Subsequently, the medium containing planktonic cells was aspirated from the wells and the latter were rinsed twice with 150 µL of phosphate buffered saline (PBS) to remove any non-adherent cells. After washing, each well was filled with PBS supplemented with different two-fold serial dilutions of compounds **7**, **26**, and **27** (from 256 to 8 µM). For comparison, a conventional antibiotic (i.e., ciprofloxacin) was tested. The plate was then incubated for 2 h at 37 °C and, after treatment, the wells were rinsed twice with PBS, as indicated above. Aliquots of 150 µL of MTT (0.5 mg/mL) were dispensed in each well to evaluate biofilm cell viability after 4 h incubation at 37 °C. This colorimetric assay consisted in the conversion of the water-soluble yellow dye MTT to the insoluble purple formazan crystals by dehydrogenases. The higher intensity of purple color corresponds to a higher percentage of metabolically active cells and consequently to a higher cell viability. The reaction was stopped by adding sodium dodecyl sulfate (SDS) (at a final concentration of 5% *v*/*v*) and the absorbance of each well was recorded at 570 nm using a microplate reader (Infinite M200; Tecan, Salzburg, Austria). The percentage of biofilm viability was calculated with respect to the untreated samples.

### 4.6. Cytotoxicity Assays

Compounds **7**, **26**, and **27** were assayed for their potential in vitro toxicity against human keratinocytes (HaCaT cells), as already described in [67]. Cells resuspended in DMEMg supplemented with 2% FBS were seeded at a density of 4 × 10^4^ per well in a 96-well plate and were incubated at 37 °C in a 5% CO_2_ atmosphere. After overnight incubation, the cells were treated with compounds at the indicated concentrations for 24 h. Controls were HaCaT cells treated with the vehicle. Afterwards, the medium of each well was replaced by 0.5 mg/mL of MTT in Hank’s buffer and the plate was incubated for further 4 h at 37 °C and 5% CO_2_. Acidified isopropanol was added to each well and absorbance was measured at 570 nm by the same microplate reader employed for the antimicrobial assays. The percentage of cell viability was calculated with respect to the control cells. The values of LC_50_ and ABC_50_ were calculated using the “Quest Graph™ LC_50_ Calculator (AAT Bioquest, Inc., Sunnyvale, CA, USA, Access date: 19 October 2021, https://www.aatbio.com/tools/lc50-calculator).

## Figures and Tables

**Table 1:** List of the molecules tested in this work and their general features.

Cluster	Common Name (Library Code)	Chemical Structure	M.W.	Molecular Formula	Source	Ref
**Alkaloids**						
**1**	Ibogaine (BBN236)	* 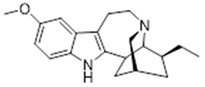 *	310.43	C_20_H_26_N_2_O	*Tabernanthe iboga* (Apocynaceae family)	[16]
**2**	Serotonin (BBN187)	* 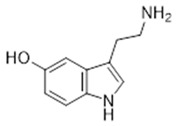 *	176.22	C_10_H_12_N_2_O	*Mucuna pruriens* (Fabaceae family); *Musa sapientum* (Musaceae family); *pineapple* (Bromeliaceae family); *strawberry and cherries* (Rosaceae family); *rice* (Poaceae family)	[17]
**3**	Caffeine (BBN195)	* 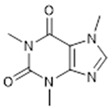 *	194.19	C_8_H_10_N_4_O_2_	*Camellia sinensis* (Theaceae family); *Coffea arabica* (Rubiaceae family)	[18]
**Phenolic compounds**						
**Aromatic compounds**						
**4**	Veratric Acid (BBN227)	* 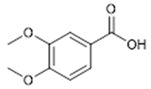 *	182.18	C_9_H_10_O_4_	*Tabebuia impetiginosa* (Bignoniaceae family)	[19]
**5**	Cinnamic Acid (BBN232)	* 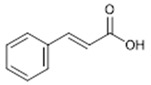 *	148.16	C_9_H_8_O_2_	*Ginseng* (Araliacee family), *Xuanshen* (Scrophulariaceae family); *Danshen* (Lamiaceae family)	[20]
**6**	Gallic Acid (BBN241)	* 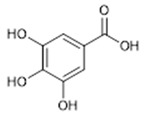 *	170.12	C_7_H_6_O_5_	*Terminalia chebula* (Combretaceae family)	[21]
**Anthranoids**						
**7**	Ferruginin A (BBN240)	* 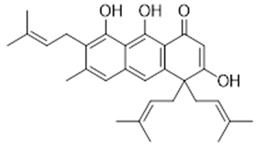 *	460.61	C_30_H_36_O_4_	*Vismia baccifera* var. *ferruginea* and *Vismia decipiens* (Hypericaceae family)	[22]
**8**	Trachyphone (BBN242)	* 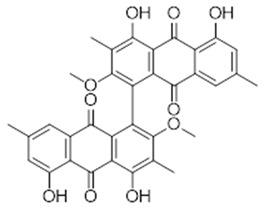 *	594.6	C_34_H_26_O_10_	*Cassia trachypus* (Leguminosae family)	[23]
**9**	Aloin (BBN36)	* 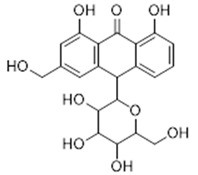 *	418.40	C_21_H_22_O_9_	*Aloe vera* (Asphodelaceae family)	[24]
**Flavonoids**						
**Rotenoids**						
**10**	Deguelin (BBN238)	* 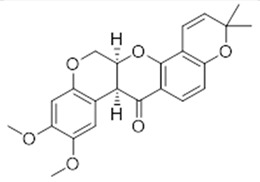 *	394.42	C_23_H_22_O_6_	*Tephrosia vogelii* (Fabaceae family)	[25]
**Furanoflavones**						
**11**	Pongapin (BBN259)	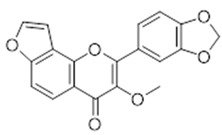	336.3	C_19_H_12_O_6_	*Pongapia pinnata* (Fabaceae family)	[26]
**Flavones**						
**12**	7-hydroxy-flavone (BBN143)	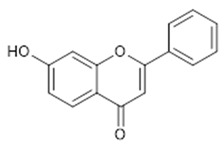	238.24	C_15_H_10_O_3_	*Tecoma stans* (Bignoniaceae family) *Ficus carica* Linn. (Moraceae family); *Oxytropis falcata* (Fabaceae family) *Clerodendrum phlomidis* (Lamiaceae family)	[27,28,29,30]
**Isoflavones**						
**13**	Glabrescione B (BBN234)	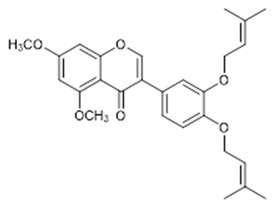	450.53	C_27_H_30_O_6_	*Derris glabrescens* (Leguminosae family)	[31]
**14**	Osajin (BBN98)	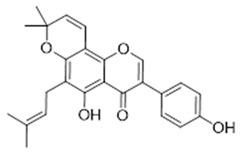	404.46	C_25_H_24_O_5_	*Maclura pomifera* (Moraceae family); *Millettia pulchra* (Leguminosae family); *Deguelia* genus (Fabaceae family)	[32,33,34]
**Flavanon**						
**15**	Sakuranetin (BBN159)	* 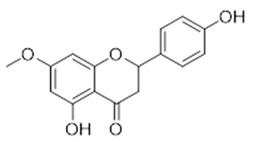 *	286.28	C_16_H_14_O_5_	*Prunus puddum* (Rosaceae family) *Prunus* *spp.* (Rosaceae family); *Baccharis retusa* (Asteraceae family); *Ribes nigrum L.* (Grossulariaceae family); *Iris milesii* (Iridaceae family); *Eriodictyon californicum* (Boraginaceae family); *Hyptis salzmanii* (Lamiaceae family); *Bonnetia dinizii* (Guttiferae family); *Primula sieboldii* (Primulaceae family); *Prunus avium* L. (Rosaceae family)	[35]
**Benzophenone**						
**16**	Clusiacitran B (BBN38)	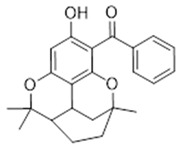	364.44	C_23_H_24_O_4_	*Clusia multiflora* (Clusiaceae family)	[36]
**Dibenzofuran**						
**17**	Usnic acid (BBN66)	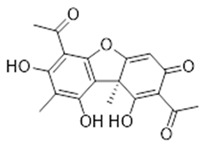	344.32	C_18_H_16_O_7_	*Ramalina hierensis* (Ramalinaceae family)	[37]
**Chalcone**						
**18**	2’,4-hydroxy-4’-methoxy-chalcone (BBN246)	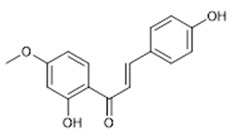	270.28	C_16_H_14_O_4_	Synthetic origin	[14]
**19**	4,4’-dimethoxy-chalcone (BBN229)	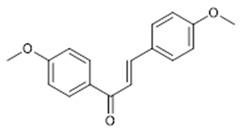	268.31	C_17_H_16_O_3_	*Angelica keiskei koidzumi* (Apiaceae family)	[38]
**Dihydrochalcone**						
**20**	2-hydroxy-dihydrochalcone (BBN86)	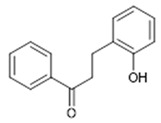	226.28	C_15_H_14_O_2_	Synthetic origin	[39]
**Coumarine**						
**21**	Xanthotoxin (BBN225)	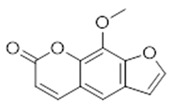	216.19	C_12_H_8_O_4_	*Ammi majus* and *Heracleum* genus (Apiaceae family)	[40,41,42]
**22**	Columbianetin (BBN133)	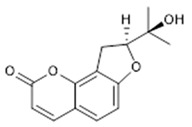	246.26	C_14_H_14_O_4_	*Angelica komarovii* (Apiaceae family); *Campylotropis hirtella* (Onagraceae family); *Melicope semecarpifolia* and *Phebalium stenophyllum* (Rutaceae family)	[43,44,45,46]
**Terpenoids**						
**23**	Borneol (BBN245)	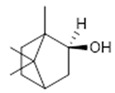	154.25	C_10_H_18_O	*Kaempferia galanga* (Zingiberaceae family); *Blumea balsamifera and Artemisia* genus (Asteraceae family)	[47]
**24**	Ursolic Acid (BBN233)	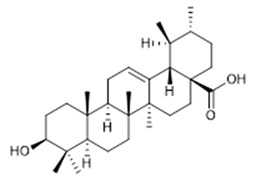	456.71	C_30_H_48_O_3_	*Mimusops* *caffra* (Sapotaceae family); *Ilex paraguarieni* (Aquifoliaceae family); *Glechoma hederaceaes* (Lamiaceae family); *Ichnocarpus frutescens* (Apocynaceae family); *Syzygium claviflorum* (Myrtaceae family)	[48,49,50]
**Apocarotenoid**						
**25**	Bixin (BBN103)	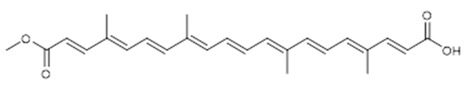	394.51	C_25_H_30_O_4_	*Bixa orellana* (Bixaceae family)	[51]

**Table 2:** Diameters of the inhibition zone of all the active compounds (15 nmol) against two Gram-positive bacterial strains.

Compound	Inhibition Zone (cm)	
	Gram-positive	
	*S. aureus*	*S. epidermidis*
**6**	2.350	n.a.
**7**	0.620	0.550
**17**	1.040	2.832
**18**	0.622	0.590
**21**	0.420	n.a.
**24**	1.040	2.832

Data are from a single experiment representative of three independent experiments; n.a.: not active.

**Table 3:** General features of the two structural related anthranoids, i.e., **26** and **27**.

Compound	Common Name (Library Code)	Chemical Structure	M.W.	Molecular Formula	Source	Ref
**26**	Ferruanthrone (BBN257)	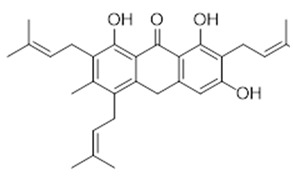	460.61	C_30_H_36_O_4_	*Vismia baccifera* var. *ferruginea* and *Vismia decipiens* (Hypericaceae family)	[22]
**27**	Vismione B (BBN239)	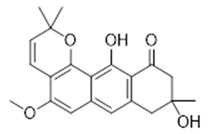	354.40	C_21_H_22_O_5_	*Vismia baccifera* var. *dealdata* (Hypericaceae family)	[55]

**Table 4:** Antimicrobial activity of **7**, **26**, and **27** against a panel of Gram-negative and Gram-positive bacteria, and yeast.

Microorganism	MIC (μM)		
	7	26	27
**Gram-negative**			
*E. coli* ATCC 25922	>256	>256	>256
*P. aeruginosa* ATCC 27853	>256	>256	>256
**Gram-positive**			
*B. megaterium* Bm11	8	256	>256
*S. aureus* ATCC 25923	64	>256	> 256
*S. epidermidis* ATCC 12228	16	>256	>256
**Yeast**			
*C. albicans* ATCC 24433	>256	>256	>256

**Table 5:** LC_50_, ABC_50_, and TI of compounds **7** and **26**.

Compound	LC_50_ (μM)	ABC_50_ (μM)		TI (LC_50_/ABC_50_)
		*S. aureus*	*S. epidermidis*	
**7**	27.88	1.08	4.5	25.81–6.19
**26**	133.53	501.8	50.32	0.26–2.65

LC_50,_ lethal concentration 50; ABC_50_, anti-biofilm concentration 50; TI, therapeutic index.
